# Mycoviruses in the Plant Pathogen *Ustilaginoidea virens* Are Not Correlated with the Genetic Backgrounds of Its Hosts

**DOI:** 10.3390/ijms18050963

**Published:** 2017-05-03

**Authors:** Jie Zhong, Chuan Yuan Cheng, Bi Da Gao, Qian Zhou, Hong Jian Zhu

**Affiliations:** 1 Hunan Provincial Key Laboratory for Biology and Control of Plant Diseases and Insect Pests, Hunan Agricultural University, Nongda Road 1, Furong District, Changsha 410128, China; wzzhtx@sina.com (J.Z.); ccy479983454@sina.com (C.Y.C.); bdgao@aliyun.com (B.D.G.); 2 Southern Regional Collaborative Innovation Center for Grain and Oil Crops in China, Hunan Agricultural University, Nongda Road 1, Furong District, Changsha 410128, China

**Keywords:** mycovirus, dsRNA, *Ustilaginoidea virens*

## Abstract

*Ustilaginoidea virens*, the causal agent of rice false smut, is one of the most devastating grain diseases that causes loss of yield in most rice-growing areas worldwide. In this study, we performed a dsRNA screen to isolate mycoviruses from 35 *U. virens* strains. The results revealed that 34 of the tested isolates were infected by various dsRNA elements, displaying highly viral diversity and mixed infections. We characterized a 5.3 kbp dsRNA from a typical isolate containing dsRNA segments with sizes ranging from 0.5 to 5.3 kbp. Sequence analysis of its genomic properties indicated that it is a novel victorivirus, named *Ustilaginoidea virens* RNA virus 5 (UvRV5), that belongs to the family *Totiviridae*. RT-PCR detection was performed and indicated that not all the dsRNA bands that were 5.3 kbp in size contained UvRV5. Moreover, the genetic relatedness of all the *U. virens* strains was estimated according to phylogenetic analysis of the partial intergenic spacer region (IGS) sequences. However, concordance was not found between the dsRNA profiles and the IGS-based genetic relatedness of their host fungi.

## 1. Introduction

*Ustilaginoidea virens*, which was first reported in the Tirunelveli district of Tamil Nadu State in India, is the causal agent of false smut disease in rice [1]. False smut can result in lost yield in almost all rice-growing areas of the world and has developed to be one of the most devastating grain diseases due to hybrid rice becoming widely planted [2]. *U. virens*-infected rice spikelets form yellow, orange, green or greenish-black smut balls after this pathogenic fungus colonizes rice floral organs, which are initiated via a small gap between the lemma and palea followed by filaments [3,4]. In addition, the false smut balls that contaminate rice panicles often contain ustiloxin, an inhibitor of microtubules [5], and are poisonous to humans and animals [6].

Mycoviruses (also called fungal viruses) are viruses that are widely distributed in almost all fungal groups, including filamentous fungi and yeasts [7,8]. While there have been single reported cases of single-stranded DNA (ssDNA) and negative single-stranded RNA (−ssRNA) mycoviruses [9,10], the genomes of most mycoviruses that have been reported so far are composed of either double-stranded RNA (dsRNA) or positive, single-stranded RNA (+ssRNA). DsRNA viruses are often encapsidated in isometric particles and are currently classified into six families: *Totiviridae*, *Partitiviridae*, *Megabirnaviridae*, *Chrysoviridae*, *Quadriviridae* and *Reoviridae* [11]. Mycoviruses with single-stranded RNA genomes are classified into seven families: *Alphaflexiviridae*, *Barnaviride*, *Endornaviridae*, *Gammaflexiviridae*, *Hypoviridae*, *Narnaviridae*, and the newly proposed family *Fusariviridae*.

Usually, mycoviruses cause latent or cryptic infection, and their fungal hosts show no obvious symptoms [7]; however, some mycoviruses lead to visible, debilitating symptoms in their host fungi, and these have been suggested as potential agents for controlling fungal diseases. To date, mycoviruses in the families of *Hypoviridae*, *Megabirnaviridae*, *Narnaviridae*, *Partitiviridae* and *Reoviridae*, as well as other viral taxa, including −ssRNA and ssDNA mycoviruses, have been identified to cause hypovirulence in their host fungi. The fact that Cryphonectria hypovirus 1 successfully controlled chestnut blight in Europe, together with the constant reports of the increasing number of novel mycoviruses that have shown some unique molecular and biological features and taxonomic considerations, has inspired pathologists to research mycoviruses [7,9,10,12,13,14].

dsRNA elements, indicative of viral infections, have been reported in some *U. virens* strains. These dsRNAs often occur as mixed infections, including viruses in the *Partitiviridae* and *Totiviridae* families. For example, the *Ustilaginoidea virens* RNA virus 1 (UvRV1) and the *Ustilaginoidea virens* partitivirus 1(UvPV1) that simultaneously occurred in a single *U. virens* strain, JYH-ZT, were first reported in this fungus [15,16]. Four viruses, *Ustilaginoidea virens* partitivirus 2 (UvPV2) [17], *Ustilaginoidea virens* RNA virus 3 (UvRV3) [18], *Ustilaginoidea virens* RNA virus 2 (UvRV2) and *Ustilaginoidea virens* partitivirus 4 (UvPV4) [19], as well as a non-segmented virus [20], have been found. In addition, a *Ustilaginoidea virens* partitivirus 3 (UvPV3), formerly named UvPV2 [21], and an unassigned virus named *Ustilaginoidea virens* non-segmented virus (UvNV-1), which is closely related to Bryopsis mitochondria-associated dsRNA, have been reported [22]. These findings indicate that *U. virens* has a repertoire of mycoviruses with substantial numbers and high diversity from which we can gain insights into the evolution and ecology of viruses as well as gain a better understanding of mycovirus-host interactions.

In this paper, we reported a novel mycovirus, designated *Ustilaginoidea virens* RNA virus 5 (UvRV5), from a *U. virens* strain and investigated whether there is a correlation between dsRNA profiles and the genetic relationship of their host fungi.

## 2. Results

### 2.1. dsRNA Elements Were Prevalent in Most of the Screened U. virens Strains

For this study, 35 *U. virens* strains were screened for the presence of dsRNAs. The prevalence of dsRNA infection in this fungal species was very high, with 34 tested strains containing dsRNAs in various sizes (Figure S1). The information for the *U. virens* strains is shown in Figure S2.

**Figure 1:**
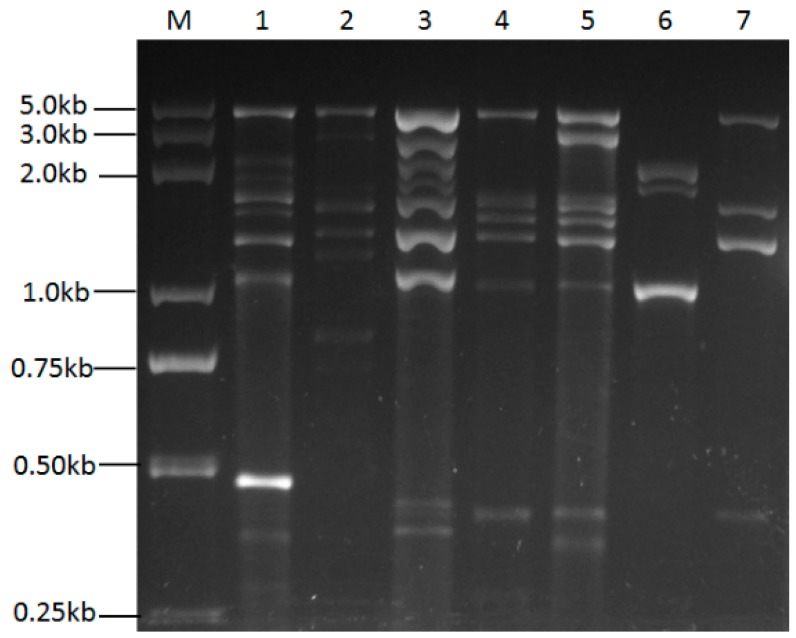
Agarose gel electrophoresis of dsRNAs extracted from several strains of *U. virens.* Lane M: 5 kbp ladder DNA marker; Lanes 1–7: FNH1212, Fyj1236, FNH1225, F10-15, F10-338, F10-341, F10-336.

As evidenced from the dsRNA banding pattern in Table 1, different fungal isolates harbored a diversity of dsRNA bands, with estimated sizes ranging from 0.25 to 5.3 kbp. Some isolates contained as many as 11 dsRNA segments. Among these, some dsRNA segments that had similar sizes were common among different strains. Five major dsRNA combinations could be deduced from the dsRNA electrophoresis patterns (Table 1): the first group consisted of three dsRNA molecules that were 5.3, 1.5 and 1.4 kbp in size; the second group included 9 to 11 dsRNA elements ranging in size from 0.25 to 5.3 kbp; the third group was a combination of only 2 dsRNA segments whose sizes were 1.5 and 1.4 kbp, respectively; the fourth group consisted of only 4 to 5 dsRNA elements ranging in size from 1 to 2.5 kbp; and the fifth group included only one dsRNA segment that was 5.3 kbp in size. Notably, an approximately 5.3 kbp dsRNA segment was widespread in most of the infected strains, and it often co-existed with other dsRNA elements. Electrophoretic profiles of dsRNA elements from some *U. virens* strains are shown in Figure 1. According to the dsRNA segment combinations and the dsRNA banding pattern, we can speculate the existence of mixed viral infection in these *U. virens* strains.

### 2.2. Sequence Analysis of a Novel dsRNA Mycovirus in Strain F10-338

**Figure 2:**
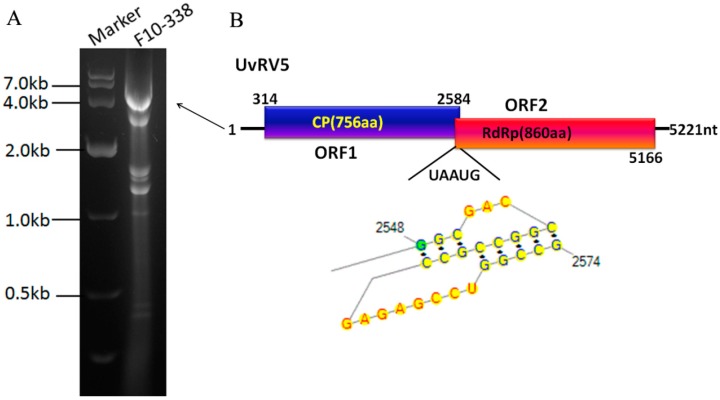
Electrophoresis and genome organization of UvRV5. (**A**) dsRNA banding pattern isolated from the *U. virens* strain F10-338 on a 1% agarose gel; (**B**) Genome organization of UvRV5. The genome of UvRV5 is 5221 nt in length and contains two overlapped open reading frames (ORF1 and ORF2), each of which encode a putative CP and RdRp, respectively. The sizes of the 5′-UTR and 3′-UTR are indicated above the solid line. The two ORFs are indicated by color boxes. A H-type pseudoknot structure consisting of stems and loops was predicted which were indicated by black and red characters, respectively.

Some dsRNA segments with sizes ranging from 0.25 to 5.3 kbp were repeatedly found in the *U. virens* strain F10-338 (Figure 2A). Among these, the largest 5.3 kbp segment, which had a higher concentration compared to other dsRNA segments according to the gel pictures, was completely sequenced. In addition, other segments were partially sequenced, which favors the idea that these dsRNA segments in strain F10-338 are present as mixed infections.

The complete sequence of the 5.3 kbp dsRNA was determined to be 5221 nt in length, with a GC content of 61.3%. It contains two overlapping open reading frames (ORF1 and ORF2): ORF1, initiating at nt position 314 and terminating at a UAA codon at nt position 2584, encoded a 756-aa protein with a molecular mass of 79.2 kDa; ORF2 (from 2584 to 5166 nt) was predicted to encode a 93.4 kDa protein composed of 860 aa (Figure 2B). A pentanucleotide, UAAUG, which constituted the stop codon of ORF 1 (UAA) and the start codon (AUG) of ORF2, was found at the nucleotide positions 2582 to 2586. Previously observed in victoriviruses, an H-type pseudoknot, which has been confirmed to be involved in the reinitiation of the translation of downstream RdRp (Li et al., 2011), was detected in seven nucleotides upstream of the UAAUG pentanucleotide and is shown as follows (bold and italic lowercase letters): *ggc*GAC*cggcc*gccGAGAGCCU*ggccg*CGCCGAA (UAAUG) (Figure 2B). An Ala/Gly/Pro-rich region was present at the C-terminus of the putative capsid proteins (CP), which is commonly found in the Victorivirus genus of the family *Totiviridae* [23].

**Figure 3:**
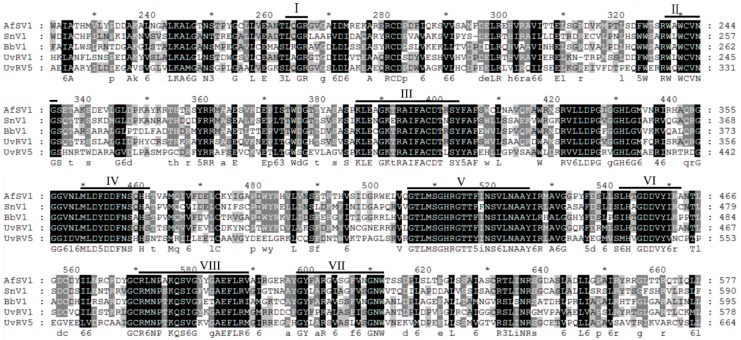
Multiple alignments of the amino acid (aa) sequences of the RdRp protein of UvRV5 and other related victoriviruses in the family *Totiviridae*. The conserved motifs of RdRp in dsRNA viruses are indicated by Roman numerals. The black and grey colors respectively mean that the amino acids were completely and partial conservative.

A homology search with BLASTp showed that the deduced amino acid (aa) sequences of ORF1 and ORF2 exhibited a high degree of identity to those of the CP and to the RNA-dependent RNA polymerase (RdRp), respectively, of viruses in the family *Totiviridae*, with the Aspergillus foetidus slow virus 1 (AfsV1) being the best matched (aa identities of 41% for the CP and 41% for the RdRp) (Table 2). GenBank accession numbers and acronyms for mycoviruses used in the analyses are listed in Table 3. In addition, a conserved viral RdRp domain with eight conserved motifs that are characteristic of RdRps from dsRNA mycoviruses that infect lower eukaryotes was found in the ORF2-encoded protein according to a conserved domain database search and multiple protein alignment analysis (Figure 3). Thus, we can suggest this dsRNA segment to be a genomic component of a novel victorivirus in the family *Totiviridae*, and we have tentatively named it *Ustilaginoidea virens* RNA virus 5 (UvRV5). The accession number for UvRV5 is KT188753.1.

### 2.3. Phylogenetic Analysis of UvRV5

**Figure 4:**
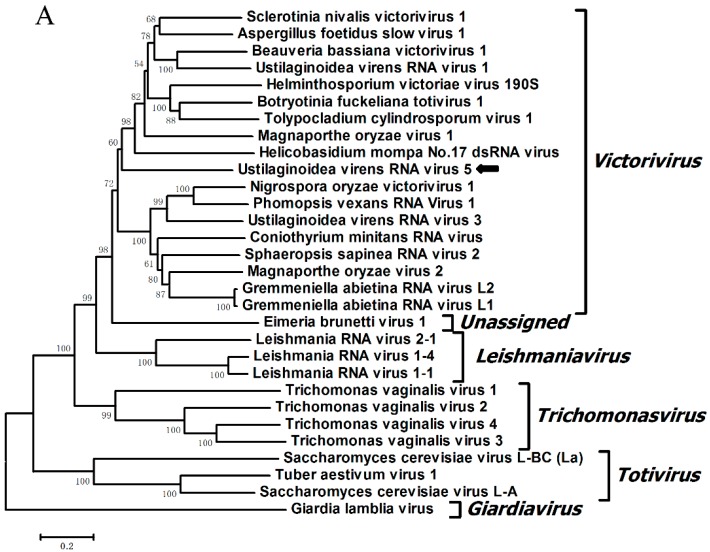

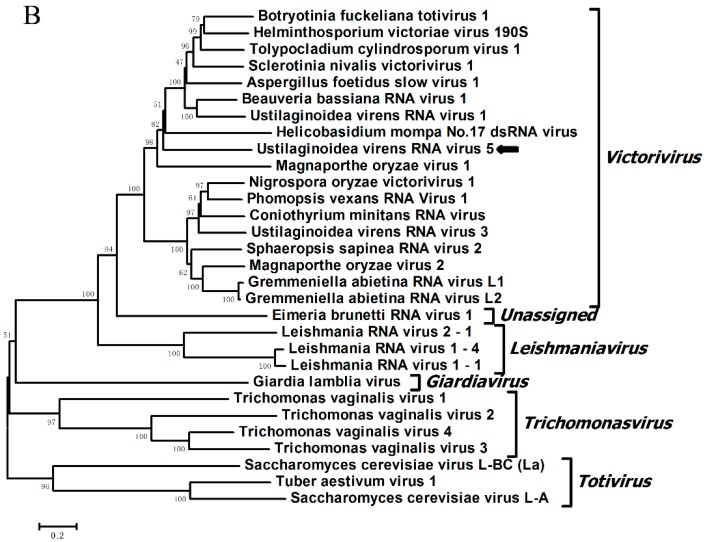
Phylogenetic analysis of UvRV5. The neighbor-joining phylogenetic trees were respectively constructed by MEGA6 with 1000 bootstrap replicates based on the aa sequences of putative RdRp and CP of UvRV5 and other members of the family *Totiviridae*. The values at nodes indicate the percentage of bootstrap replicates supporting the branch. The UvRV5 was indicated by black arrows in the phylogenetic trees. The virus names and accession numbers used are shown in Table 3.

The neighbor joining (NJ) phylogenetic trees were constructed based on the RdRp and CP domains of UvRV5 and other representative members of the family *Totiviridae*. RdRp-based phylogenetic analysis, as shown in Figure 4, indicated that UvRV5 clustered with members of the genera *Victorivirus* and separated from the members of genera *Leishmaniavirus*, *Trichomonasvirus*, *Totivirus*, and *Giardiavirus*. In addition, the topologies between the CP- and RdRp-based phylogenetic trees were similar, further supporting the taxonomic status of the UvRV5 virus.

### 2.4. Phylogenetic Analysis of IGS Sequences

**Figure 5:**
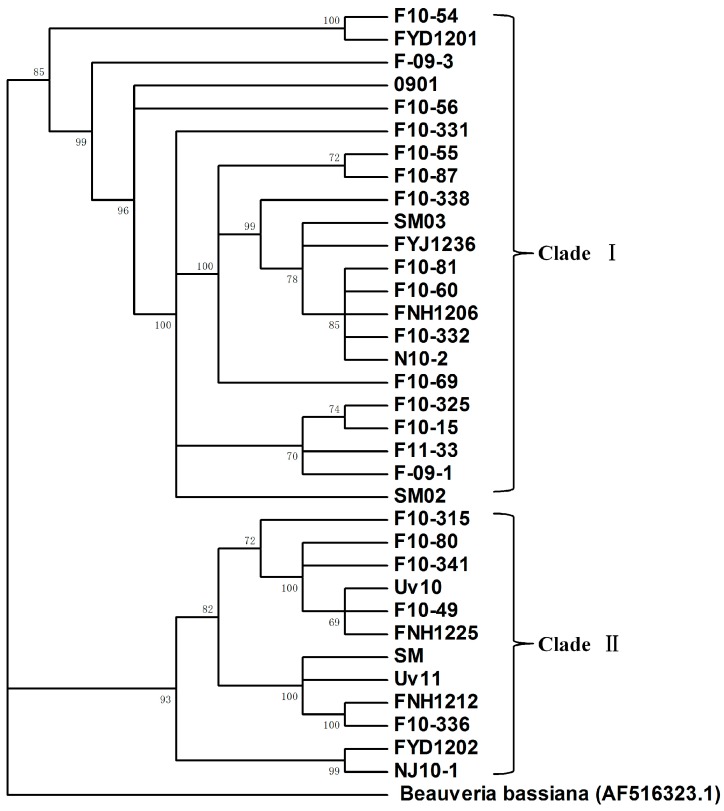
Maximum likelihood phylogenetic tree generated from the partial IGS rDNA region sequences of the *U. virens* strains. The *Beauveria bassiana* strain (AF516323.1) was included as an outgroup. The phylogenetic tree clustered the 34 *U. virens* strains into two main clades (Clade I and II).

To study the genetic relationship of the dsRNA-containing *U. virens* strains, we amplified and sequenced their partial IGS sequences. Phylogenetic analysis inferred by PhyML 3.0 using the ML method demonstrated that these 34 *U. virens* strains could be divided into two main clades, clade I and II. Clades I and II had 22 and 12 isolates, respectively (Figure 5).

### 2.5. dsRNA Infection is Not Genetically Related to the Host Fungi

Since *U. virens* strains are geographically widespread and are associated with high incidences of dsRNA infection, we attempted to assess whether any dsRNA profiles in the *U. virens* strains were associated with the genetic background of their host. However, phylogenetic analysis based on IGS sequences indicated that there was no concordance between similar dsRNA profiles and the genetic relationship of their host fungal strains was found. For example, isolates F10-33, F10-325 and Fyj1236, which shared similar IGS sequences and were clustered in the same clade, have different dsRNA profiles, while other *U. virens* strains belonging to different IGS clades, such as F11-33 and F10-336, contained the same dsRNA profile.

### 2.6. Detection of UvRV5 in Other U. virens Strains

**Figure 6:**
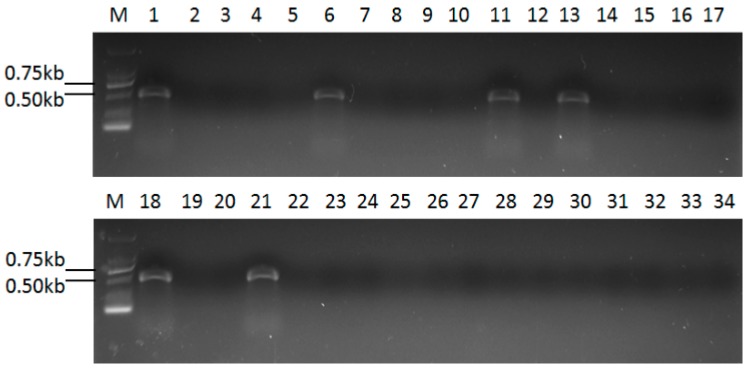
Results of RT-PCR assays in the *U. virens* strains using the primers designed from UvRV5. Segments of approximately 500 bp were amplified from six of these *U. virens* strains, which indicate infection by UvRV5 or by a UvRV5 homologue. Lane 1: F10-338 (positive control); lane 6: FNH1212; lane 11: F10-336; lane 13: Fyj1236; lane 18: F10-331; lane 21: FNH1225.

To check whether other *U. virens* strains were infected with UvRV5, RT-PCR was performed using primers designed based on this virus. The result showed that only five strains harboring the 5.3 kbp dsRNA banding pattern contained UvRV5 (Figure 6), indicating that not all the 5.3 kbp dsRNA elements that were infected in other strains belong to UvRV5, and they might be infected with other mycoviral species. Therefore, it can be assumed that the mycovirus diversity that exists in these analyzed *U. virens* strains is abundant.

## 3. Discussion

In this study, we screened dsRNA elements from a collection of 35 *U. virens* strains and reported the discovery of the genome of a novel victorivirus in a *U. virens* strain. The dsRNA element screening results revealed that mycovirus infections are commonly present among *U. virens* strains, with infection rates of 97%. The electrophoresis patterns of the dsRNAs isolated from the 34 infected *U. virens* strains displayed five major dsRNA combinations. In addition, we confirmed that dsRNA electrophoretic profiles of *U. virens* strains were not associated with their genetic backgrounds that were estimated from the partial IGS sequences.

A dsRNA combination containing a set of approximately 9 to 12 dsRNA segments that ranged in size from 0.5 to 5.3 kbp was represented by strain F10-338. We selected this strain for dsRNA sequence characterization. A dsRNA segment with an electrophoretic deduced molecular weight of 5.3 kbp, similar to those with a comparable molecular weight that existed not only in F10-338 but also in other strains, was cDNA cloned and the sequence was determined. Based on genomic organization, homology searches of the aa sequences of their CP and RdRp, the existence of characteristic motifs in RdRp and a predicted RNA pseudoknot structure located upstream of the CP stop codon, which is involved in the coupled termination reinitiation mechanism for downstream RdRp translation in victoriviruses [24], we deduced this dsRNA segment to be the genome of a novel victorivirus in the family *Totiviridae.*

As we know, mycoviruses are widespread in almost all major taxa of fungi, including filamentous fungi and yeasts, and in most cases they are associated with causing latent infections in their hosts. However, different fungal hosts might have diverse prevalence values of mycoviral infections. Extensive screens of many fungal species for the presence of dsRNA revealed that the incidence of mycoviral infection varies, with some cases even greater than 80% [7]. In our research and in accordance with previous reports [25], the incidence of dsRNA infection in *U. virens* was much higher (up to 97% in this study) than the incidence of other fungal species that were screened previously, such as *Aspergillus fumigatus* (6.6% incidence) [26], *Beauveria bassiana* (54.8% incidence) [27], *Tolypocladium cylindrosporum* [28], *Epichloë festucae* [29], *Chalara elegans* (up to 80% incidence) [30], *Cryphonectria parasitica* [31], *Rosellinia necatrix* [32,33], *Helicobasidium mompa* [33] and *Heterobasidion* sp. [34,35]. Chlamydospores and conidia are abundantly produced during the *U. virens* life cycle, and as the dominant pathway for reproduction of this pathogenic fungus, they can be efficiently dispersed. In addition, the smut balls composed of chlamydospores compounded in rice might migrate long distances by means of humans to disperse this false smut disease. Furthermore, the transmission of mycoviruses via conidiation is very effective in *U. virens* [25]. Thus, these reasons may have contributed to the high prevalence values of mycoviral infection in *U. virens*.

On the other hand, the high incidence of mycoviral provenance in *U. virens* might shed some clues about the extended period of coevolution between fungal hosts and mycoviruses to elucidate how the viruses have evolved to result in symptomless infections. Some fungal hosts infected by some debilitation/hypovirulence-associated mycoviruses often exhibit reduced mycelial growth and hypovirulence, which causes them to lose or to have weakened survival and transmission abilities [7]. Under this circumstance, it is very difficult for these viruses to spread, occur extensively and to evolve long-term among and within their host fungi. Of course, we could not exclude the possibility that some dsRNA mycoviral infections benefit their host fungi, which further contributes to the maintenance of these dsRNA mycoviruses in *U. virens.* For example, a virus named Curvularia thermal tolerance virus (CThTV) that infects a Curvularia root endophyte has been reported to enhance the thermal tolerance of the endophyte from this plant host [36]. Evidence has been provided recently that some mycoviruses might affect the endophytic capability of some fungal strains, such as the virus in *T. cylindrosporum* [37]. However, to confirm this similar possibility in *U. virens*, further studies to exactly elucidate the functions of these dsRNAs are required.

To determine the genetic diversity among the different isolates of a fungal species, diverse genetic marker systems, such as allozymes, AFLP and microsatellites, as well as multilocus sequence typing, (MLST) were often used [38,39]. The intergenic spacer (IGS) residing between the 28 and 18 S rDNA genes has high interspecific and intraspecific variability, which makes it a useful molecular tool for the phylogenetic analysis of a wide number of fungal taxa at the strain level [40,41]. In this article, we sequenced the partial IGS sequences of 34 *U. virens* strains infected by different dsRNA elements. Phylogenetic analysis based on the ML method grouped these *U. virens* strains into two main clusters. However, the clustering reflecting the genetic background of the *U. virens* was unrelated to the dsRNA banding patterns. This discordance was also reported in other studies. Refos et al. [42] analyzed dsRNA mycoviruses from a collection of 86 clinical *Aspergillus fumigatus* strains, and they found that the dsRNA banding patterns were independent of the genetic makeup of the host fungus.

Currently, knowledge of the genetic diversity of *U. virens* populations is limited, to some degree. Zhou et al. [43] analyzed the genetic diversity and population characteristics using amplified fragment length polymorphism (AFLP) markers of this pathogen. Recently, three SNP-rich genomic regions have been developed as novel molecular markers to study the population structure of *U. virens* isolated from China on a large geographical scale [44]. IGS rDNA is not the sole method for investigating genetic diversity, and it sometimes contradicts other methods that are based on other genes, as reported in studies of the *Fusarium oxysporum* species complex (FOSC) by O’Donnell et al. [45] in which there was some discordance between the IGS rDNA and the EF-1α bipartitions. Therefore, future research that focuses on gene sequences other than IGS should be performed to better understand the phylogeny between different *U. virens* strains.

It appeared that mycoviral infection of *U. virens* isolates was not related to the genetic background of their host. This indicated that mycoviruses may harbor the ability to infect all isolates of *U. virens.* On the other hand, it is reasonable to consider that the dsRNA mycoviruses infecting *U. virens* might be transmissible among isolates of the fungal host given that the dispersal processes of *U. virens* is very effective, which might provide a contiguous environment for different isolates.

## 4. Materials and Methods

### 4.1. Fungal Isolates and Growth Conditions

To analyze the prevalence of dsRNA mycoviruses in *U. virens*, 35 isolates from rice plants infected by false smut in China were used. These fungal isolates were maintained on potato sucrose agar plates. Mycelia plugs were grown in potato sucrose broth while shaking (170 rpm) at 28 °C for 7 days to harvest mycelium to be used for dsRNA isolation extraction.

### 4.2. dsRNA Extraction

dsRNAs were extracted as described by Morris and Dodds [46] using CF cellulose (Sigma, St. Louis, MO, USA). To remove contaminating DNA and ssRNAs, the extracts were subjected to RNase-free DNaseI and S1 nuclease (TaKaRa, Dalian, China) digestion. These dsRNAs were estimated for size and banding pattern by 1% agarose gel electrophoresis, and the gels were visualized with a gel imaging system after being stained with ethidium bromide. dsRNA extractions and visualization were conducted in this study three times independently. For cDNA cloning, separated dsRNA segments were purified from the agarose gel using a gel extraction kit (TaKaRa) and stored at −20 °C.

### 4.3. cDNA Synthesis, Cloning, Sequencing, and Phylogenetic Analysis

dsRNAs in the isolate F10-338 were purified and used for cDNA synthesis and molecular cloning. A cDNA library was constructed using random hexadeoxynucleotide primers (Takara) and reverse transcriptase and then cloned into the T/A cloning vector pMD18-T. Competent cells from the *Escherichia coli* strain DH5α (Takara) were transformed and screened to select for transformants that contained inserts, which were later sequenced. Gaps within the assembled sequences derived from the cDNA library were filled by reverse transcription and PCR using the primers designed according to sequences flanking the gaps. To clone the dsRNA terminal sequences, a modified method described previously was used [47] that included 3′-end adaptor ligation with T4 RNA ligase (Fermentas, Vilnius, Lithuania) and PCR amplification. All amplified DNA fragments were again cloned into the pMD-18 vector and sequenced, with each base being sequenced from at least three independent clones.

Sequences were assembled using DNAMAN. Sequence similarity searches were conducted in the NCBI database by the BLAST program [48]. Alignments and phylogenetic analysis were carried out using the CLUSTALX [49] and MEGA 6 programs [50], respectively. The NJ method was used for phylogenetic tree construction, with bootstrap test values being calculated from 1000 replications.

### 4.4. DNA Isolation

Genomic DNA of these *U. virens* strains were extracted from approximately 100 mg dry weight mycelium. The mycelium was ground in liquid nitrogen and subjected to total genomic DNA extraction using the total purification DNA kit (Trans Gen Biotech, Beijing, China) according to the method described by the manufacturer.

### 4.5. IGS Amplification, Sequencing, and Phylogenetic Analysis

The partial IGS region was amplified using Taq DNA polymerase (TaKaRa, Dalian, China) with the following primers: Uv-IGS III: 5′-GGCGAAGTTGGCGGTAAGA-3′; Uv-IGS IV: 5′-CCACCATTTCGTATCTAAGTCGG-3′ [51]. The PCR reaction was carried out according to the manufacturer’s instructions. The amplification products were subjected to gel electrophoresis and visualization under UV light with ethidium bromide staining prior to being sequenced directly.

The IGS sequences were aligned with CLUSTALX [49]. A maximum likelihood (ML) phylogenetic tree was constructed with PhyML 3.0 [52]. Branch support was calculated with a Shimodaira–Hasegawa-like (SH-like) procedure using the approximate likelihood ratio test (aLRT) [53]. The GTR substitution model was used for ML phylogenetic tree generation, and the IGS sequence from a *Beauveria bassiana* strain (AF516323.1) was used as the outgroup.

### 4.6. Detection of UvRV5 in Other U. virens Strains

The following *U. virens* strains were subjected to RT-PCR to detect the presence of UvRV5. Primers UvRV5F: 5′-AATGGCGGCTCACCTATCC-3′ and UvRV5R: 5′-GCTTGCGGCACTGTCTTGT-3′ were designed based on the genomic sequence of UvRV5. Total RNA was extracted from the mycelium of each *U. virens* strain. An RT-PCR assay was conducted using reverse transcriptase for reverse transcription and *Taq polymerase* for PCR amplification according to the manufacturer’s instructions. The PCR products were electrophoresed on a 1% agarose gel, stained with ethidium bromide, and observed by UV illumination.

## 5. Conclusions

In this study, through screening of dsRNA elements from 35 *U. virens* strains, we found that there was a highly viral diversity and mixed infections in this phytopathogenic fungus. Besides, a 5.3 kbp dsRNA in a typical isolate containing dsRNA segments with sizes ranging from 0.5 to 5.3 kbp was characterized and determined to be a novel victorivirus in the family *Totiviridae*. However, confirmed by RT-PCR detection, we found that not all the dsRNA bands that were 5.3 kbp in size were belonging to the same virus. Besides, concordance was not found between the dsRNA profiles and the genetic relatedness of their host fungus which was estimated by phylogenetic analysis of the partial IGS sequences.

## Figures and Tables

**Table 1:** Electropherotypes of dsRNA elements in *U. virens*. The diagonal lines indicate the presence of dsRNA bandings in *U. virens* strains, with the molecular size shown at the top of each column. Similar sets of dsRNA combinations present in *U. virens* strains are marked with identical colors.

Strains	DsRNA Elements Observed (kbp)																	
	0.3	0.4	0.5	0.6	0.75	1.0	1.2	1.3	1.4	1.5	1.6	2.0	2.5	2.8	3.0	4.0	5.3	IGS Clade
F10-54								**/**		**/**								Clade I
FYD1201								**/**		**/**								
F-09-3	**/**	**/**	**/**		**/**			**/**		**/**							**/**	
0901								**/**		**/**							**/**	
F10-56	**/**	**/**						**/**		**/**							**/**	
F10-331	**/**	**/**				**/**		**/**	**/**	**/**		**/**	**/**	**/**			**/**	
F10-55					**/**			**/**		**/**							**/**	
F10-87																	**/**	
F10-338	**/**	**/**				**/**		**/**	**/**	**/**	**/**				**/**		**/**	
SM03																	**/**	
FYJ1236				**/**	**/**		**/**	**/**		**/**	**/**				**/**		**/**	
F10-81					**/**			**/**		**/**							**/**	
F10-60								**/**		**/**							**/**	
FNH1206									**/**	**/**								
F10-332									**/**	**/**							**/**	
N10-2								**/**		**/**							**/**	
F10-69									**/**	**/**							**/**	
F10-325																	**/**	
F10-15		**/**				**/**		**/**	**/**	**/**							**/**	
F11-33								**/**		**/**							**/**	
F-09-01		**/**	**/**			**/**	**/**		**/**	**/**							**/**	
SM02									**/**	**/**							**/**	
F10-315									**/**	**/**							**/**	Clade II
F10-80					**/**		**/**		**/**	**/**							**/**	
F10-341						**/**						**/**	**/**	**/**				
Uv10	**/**	**/**	**/**		**/**	**/**	**/**	**/**	**/**	**/**	**/**	**/**			**/**	**/**	**/**	
F10-49								**/**		**/**							**/**	
FNH1225	**/**	**/**				**/**			**/**	**/**	**/**	**/**			**/**		**/**	
SM									**/**	**/**							**/**	
Uv11		**/**	**/**			**/**	**/**	**/**	**/**	**/**							**/**	
FNH1212		**/**	**/**			**/**	**/**		**/**	**/**	**/**	**/**	**/**				**/**	
F10-336			**/**						**/**	**/**							**/**	
FYD1202		**/**				**/**		**/**	**/**	**/**	**/**	**/**	**/**				**/**	
NJ10-1	**/**	**/**	**/**					**/**	**/**	**/**							**/**	

**Table 2:** Amino acid sequence identities of the CP and RdRp between UvRV5 and other similar victoriviruses deduced by a BLASTp search.

Virus	CP				RdRp			
	Identity (%)	*E* Value	Overlap (Positions)	Query Coverage (%)	Identity (%)	*E* Value	Overlap (Positions)	Query Coverage (%)
AfsV1	41	7 × 10^−156^	290/700	92	41	0	319/776	87
SnV1	46	3 × 10^−171^	303/656	86	40	7 × 10^−172^	322/797	90
BbV1	43	1 × 10^−169^	311/719	94	39	1 × 10^−165^	313/798	90
UvRV1	43	6 × 10^−165^	304/714	94	40	1 × 10^−164^	309/780	89
HmV1-17	40	9 × 10^−148^	255/643	84	38	2 × 10^−163^	299/790	89
MoV1	37	8 × 10^−111^	227/610	79	35	2 × 10^−147^	280/789	89
GaRV-L1	38	1 × 10^−114^	261/685	86	39	2× 10^−143^	304/786	89
GaRV-L2	38	2 × 10^−114^	260/685	86	39	3 × 10^−143^	303/786	89
MoV2	38	7 × 10^−99^	225/600	77	38	3 × 10^−140^	300/798	89
PvRV	39	9 × 10^−113^	245/628	80	37	1 × 10^−138^	289/786	88
BfTV1	43	6 × 10^−172^	279/64	84	34	4 × 10^−138^	298/873	99
TcV1	43	3 × 10^−157^	272/637	84	35	4 × 10^−138^	313/886	99
UvRV3	37	1 × 10^−99^	224/606	78	37	9 × 10^−137^	288/784	88
HvV190S	46	3 × 10^−180^	289/632	83	36	2 × 10^−136^	281/788	89
SsRV2	39	9 × 10^−105^	234/607	77	37	2 × 10^−134^	300/805	91
CmRV	38	4 × 10^−105^	228/602	78	37	5 × 10^−126^	293/783	88
NoV1	36	9 × 10^−105^	236/655	84	36	8 × 10^−126^	284/786	88

**Table 3:** Information for viruses used for sequence and phylogenetic analysis.

Genus	Virus Name	Abbreviation	Accession Number.	
			CP	RdRp
Victorivirus	*Ustilaginoidea virens* RNA virus 5	UvRV5	ALP73430.1	ALP73431.1
	Beauveria bassiana victorivirus 1	BbV1	CCC42234.1	AMQ11131.1
	Botryotinia fuckeliana totivirus 1	BfTV1	YP_001109579.1	AM491608
	Coniothyrium minitans RNA virus	CMRV	YP_392466.1	AF527633
	Gremmeniella abietina RNA virus L1	GaRV-L1	NP_624331.1	NP_624332.2
	Gremmeniella abietina RNA virus L2	GaRV-L2	YP_044806.1	YP_044807.1
	Helicobasidium mompa totivirus 1-17	HmV1-17	NP_898832.1	NP_898833.1
	Helminthosporium victoriae virus 190S	HvV190S	NP_619669.2	NP_619670.2
	Magnaporthe oryzae virus 1	MoV1	YP_122351.1	YP_122352.1
	Magnaporthe oryzae virus 2	MoV2	YP_001649205.1	AB300379
	*Ustilaginoidea virens* RNA virus 1	UvRV1	AGO04406.1	AGO04407.1
	*Ustilaginoidea virens* RNA virus 3	UvRV3	YP_009004155.1	YP_009004156.1
	Aspergillus foetidus slow virus 1	AfSV1	CCD33023.1	CCD33024.1
	Sclerotinia nivalis victorivirus 1	SnV1	YP_009259367.1	YP_009259368.1
	Nigrospora oryzae victorivirus 1	NoV1	YP_009254735.1	YP_009254736.1
	Sphaeropsis sapinea RNA virus 2	SsRV2	NP_047559.1	NP_047560.1
	Tolypocladium cylindrosporum virus 1	TcV1	YP_004089629.1	YP_004089630.1
	Phomopsis vexans RNA virus	PvRV	YP_009115491.1	YP_009115492.1
Totivirus	Saccharomyces cerevisiae virus L-A	ScV-L-A	NP_620494.1	AAA50321.1
	Tuber aestivum virus 1	TaV1	ADQ54105.1	ADQ54106.1
	Saccharomyces cerevisiae virus L-BC (La)	ScV-L-BC (La)	NP_042580.1	NP_042581.1
Trichomonasvirus	Trichomonas vaginalis virus 1	TVV1	ABC86750.1	ABC86751.1
	Trichomonas vaginalis virus 2	TVV2	AED99809.1	AAF29445.1
	Trichomonas vaginalis virus 3	TVV3	NP_659389.1	NP_659390.1
	Trichomonas vaginalis virus 4	TVV4	AED99797.1	AED99798.1
Leishmania virus	Leishmania RNA virus 1-1	LRV1-1	NP_041190.1	M92355
	Leishmania RNA virus 1-4	LRV1-4	NP_619652.1	NP_619653.1
	Leishmania RNA virus 2-1	LRV2-1	NP_043464.1	U32108
Giardiavirus	Giardia lamblia virus	GLV	NP_619551.1	NP_620070.1
Unassigned	Eimeria brunetti RNA virus 1	EbRV1	NP_108650.1	CAK02788.1

## Supplementary Materials

Supplementary materials can be found at www.mdpi.com/1422-0067/18/5/963/s1.
