# Supplementary material for: Mycoviruses in the Plant Pathogen Ustilaginoidea virens Are Not Correlated with the Genetic Backgrounds of Its Hosts
Source: Int J Mol Sci. 2017 May 3;18(5):963. doi: 10.3390/ijms18050963 (PMC5454876; doi:10.3390/ijms18050963)
Supplement: Supplementary file 1 [file ijms-18-00963-s001.pdf]

Fig.S1

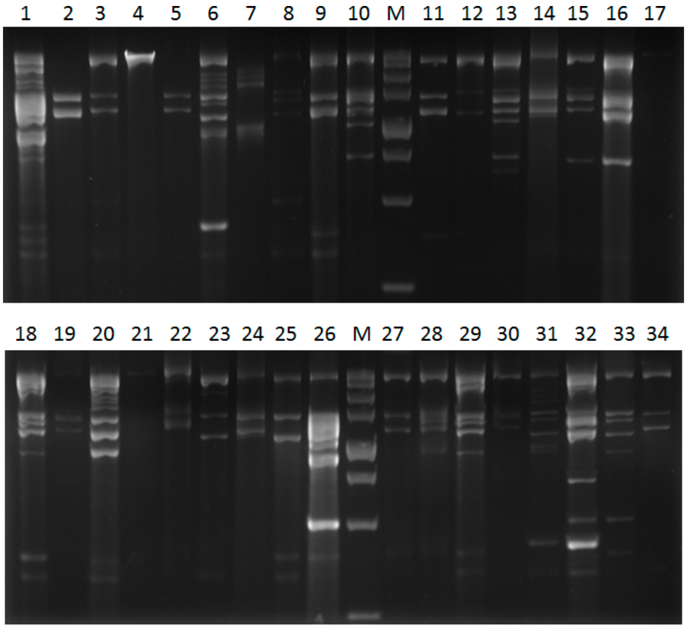

Fig.S1. (A) The electrophoretic banding patterns of dsRNA elements extracted from 34 *Ustilaginoidea virens* strains analyzed by 1% agarose gel. The numbers at the top of each lane indicated the names of these *U. virens* strains. Lanes: 1-34: Uv10; FYD1201;F11-33; F10-325;F10-54; FNH1212;F10-341; F10-340; F10-56; F10-80; F10-336; N10-2; Fyj1236; F10-60; F10-55; F10-81; F10-87; F10-331; FNH1206; FNH1225; SM03; F10-69; F10-49; SM02; 0901; Uv11; F10-315; F10-15; F10-338; F10-332; Fyd1202; F-09-3; F-09-1; SM. Lanes M means the 5 Kbp ladder DNA marker.

Fig.S2

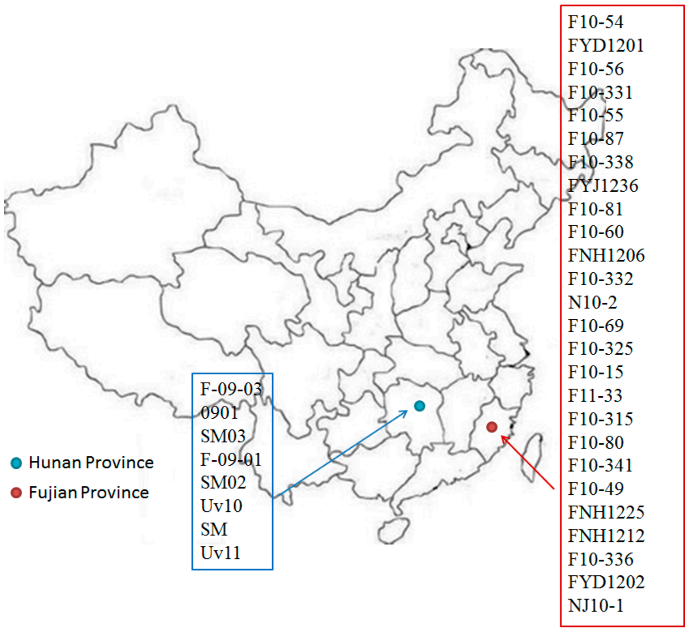

Fig.S2. Locations for collection of the *U. virens* strains. The strains were isolated from Hunan and Fujian province in china.
